# *Porphyromonas gingivalis* is the most abundant species detected in coronary and femoral arteries

**DOI:** 10.1080/20002297.2017.1281562

**Published:** 2017-02-08

**Authors:** J-L. C. Mougeot, C. B. Stevens, B. J. Paster, M. T. Brennan, P. B. Lockhart, F. K. B Mougeot

**Affiliations:** ^a^ Department of Oral Medicine, Cannon Research Center, Carolinas HealthCare System, Charlotte, NC, USA; ^b^ Department of Microbiology, The Forsyth Institute, Cambridge, MA, USA; ^c^ Department of Oral Medicine, Infection and Immunity, Harvard School of Dental Medicine, Boston, MA, USA

**Keywords:** Oral microbiome, metagenomics, HOMI*NGS*, atherosclerosis, *P. gingivalis*

## Abstract

An association between oral bacteria and atherosclerosis has been postulated. A limited number of studies have used 16S RNA gene sequencing-based metagenomics approaches to identify bacteria at the species level from atherosclerotic plaques in arterial walls. The objective of this study was to establish detailed oral microbiome profiles, at both genus and species level, of clinically healthy coronary and femoral artery tissues from patients with atherosclerosis. Tissue specimens were taken from clinically non-atherosclerotic areas of coronary or femoral arteries used for attachment of bypass grafts in 42 patients with atherosclerotic cardiovascular disease. Bacterial DNA was sequenced using the MiSeq platform, and sequence reads were screened *in silico* for nearly 600 oral species using the HOMI*NGS* ProbeSeq species identification program. The number of sequence reads matched to species or genera were used for statistical analyses. A total of 230 and 118 species were detected in coronary and femoral arteries, respectively. Unidentified species detected by genus-specific probes consisted of 45 and 30 genera in coronary and in femoral artery tissues, respectively. Overall, 245 species belonging to 95 genera were detected in coronary and femoral arteries combined. The most abundant species were *Porphyromonas gingivalis*, *Enterococcus faecalis*, and *Finegoldia magna* based on species probes. *Porphyromonas*, *Escherichia, Staphylococcus*, *Pseudomonas*, and *Streptococcus* genera represented 88.5% mean relative abundance based on combined species and genus probe detections. *Porphyromonas* was significantly more abundant than *Escherichia* (i.e. 46.8% vs. 19.3%; *p* = 0.0005). This study provides insight into the presence and types of oral microbiome bacterial species found in clinically non-atherosclerotic arteries.

An association between oral bacteria and atherosclerosis has been postulated [1–4]. Epidemiological studies have shown an association between periodontal pathogens, namely *Aggregatibacter actinomycetemcomitans* (previously *Actinobacillus actinomycetemcomitans*) and *Porphyromonas gingivalis*, and coronary artery disease and stroke. Proposed mechanisms include: (1) a distant site colonization by oral microbiota, resulting in heightened immune reactions from various origins (e.g. increase in proinflammatory cytokines), and (2) generation of cross-reactive antibodies against periodontal pathogens [5–8]. Additionally, although many studies have shown the relationship between various gingival tissue manipulations and bacteremia of oral species [9], the precise mechanisms by which oral periodontal pathogens colonize and proliferate in human artery walls has not yet been elucidated [5–8,10,11].

Metagenomics studies have largely characterized the nature of the oral microbiome in dental plaque associated with periodontal disease [12,13], so that comprehensive correspondence with bacterial profiles of arteries from atherosclerotic patients may be established in downstream investigations.

Scientific evidence supporting a possible role of oral bacterial species in atherosclerosis relies to a large extent on the detection and identification of bacterial DNA in human arterial wall tissues or atherosclerotic plaque in cross-sectional study designs [14]. Most importantly, Kozarov et al. demonstrated that viable *A. actinomycetemcomitans* and *P. gingivalis* could be isolated from atherosclerotic plaque [10].

Several metagenomics studies, using different technologies, have determined bacterial profiles in atherosclerotic plaque. These studies, however, have provided limited information at the species level or have shown little overlap at the species level with regard to the listings of taxa described [15–19]. Only one of these studies describes the presence of *A. actinomycetemcomitans* in atherosclerotic plaque [18], but none of them reported finding *P. gingivalis*. Another shortfall is the lack of confirmatory studies to determine the absolute and relative abundance for some of the more predominant species that had previously been detected by other techniques (e.g. culture, microscopy, targeted polymerase chain reaction) in arterial tissues from atherosclerotic patients and controls [14].

Confirmatory studies are thus needed to determine the number and abundance of species present in atherosclerotic plaque and clinically non-atherosclerotic artery walls of atherosclerotic patients, in particular those species associated with periodontal disease. The purpose of this study was to establish detailed oral microbiome profiles for genera and species found within clinically non-atherosclerotic coronary and femoral artery tissues using Human Oral Microbe Identification applying *N*ext *G*eneration *S*equencing (HOMI*NGS*) [20].

## Methods

### Patient population

Full-thickness tissue specimens were taken from clinically non-atherosclerotic healthy areas of two different arteries used for attachment of bypass grafts. The designation of ‘clinically non-atherosclerotic healthy’ areas is based on the clinician surgeon’s judgment made during macroscopic selection of an area to attach the graft, such area having no obvious atherosclerotic pathology. Coronary artery tissue from patients with atherosclerotic cardiovascular disease (CVD; *n* = 32) and femoral artery tissue from patients with atherosclerotic vascular disease-related blockages distant to the site of tissue acquisition vascular diseases (*n* = 10) were collected between 2002 and 2003 at the authors’ hospital through a previous Institutional Review Board–approved study. A waiver of informed consent was obtained in 2014 for this non-interventional retrospective study on de-identified specimens, involving minimal risk and appropriate PHI protection. A total of 23 coronary and eight femoral artery specimens were collected from the male cohort (*n* = 31), and nine coronary and two femoral artery specimens were collected from the female cohort (*n* = 11). Demographic information (age, sex, and ethnicity) were collected for all patients. Systolic and diastolic blood pressure information was collected for all patients except for four males. Body mass index, diabetes status, lipids panel, and C-reactive protein levels data were collected for 33, 33, 17, and 17 patients, respectively (see Table 1). Periodontal disease measurements and tissue histology data related to inflammation were not collected.

**Table 1.** Demographic and clinical characteristics.

**Characteristics**	**Data**	**Subjects included**
Age (years), *M* (*SD*)	64 (9.86)	All (*n* = 42)
Males, *n* (%); age (years), *M* (*SD*)	31 (73.8%); 62.3 (9.46)	
Females, *n* (%); age (years), *M* (*SD*)	11 (26.2%); 68.7 (9.84)	
Ethnicity, *n* (%)		Subset (*n* = 41)
White	38 (90.3%)	
African American	3 (7.3%)	
Hispanic	1 (2.4%)	
Other	0 (0%)	
SBP, *M* (*SD*), range	133.29 (25.37), 100–190	Subset (*n* = 38)
DBP, *M* (*SD*), range	71.87 (13.40), 48–100	Subset (*n* = 38)
MAP, *M* (*SD*), range	92.34 (14.56), 67.33–120	Subset (*n* = 38)
BMI, *M* (*SD*), range	27.93 (4.75), 16.18–38.55	Subset (*n* = 33)
Diabetes, *n* (%)	12 (36.4%)	Subset (*n* = 33)
Lipids panel, *M* (*SD*), range		Subset (*n* = 17)
Cholesterol	184.76 (52.25), 119–337	
Triglycerides	184 (119.72), 64–478	
HDL	105.59 (42.16), 62–242	
C-reactive protein, *M* (*SD*), range	42.41 (15.65), 27–91	Subset (*n* = 17)

Clinical characteristics relevant to atherosclerosis were obtained for patients for whom clinically healthy coronary and femoral artery tissues were recovered from bypass surgery and used for microbiome profile determination by HOMI*NGS*. Males and females were of a similar age (*M* [*SD*]). Available data indicated that based on mean values, the patient cohort was characterized by prehypertension systolic blood pressure (SBP; mmHg) between 120 and 140, normal diastolic blood pressure (DBP; mmHg) <80, high metric body mass index in ‘overweight’ category (BMI; kg/m^2^), cholesterol levels below borderline high threshold of 200 mg/dL, borderline high triglycerides (mg/dL) between 150 and 200, near optimal low density lipoproteins (LDL; mg/dL), and levels of C-reactive protein indicative of active inflammation (>10 mg/L).

HDL, high-density lipoprotein; MAP, mean arterial pressure; *SD*, standard deviation.

### HOMINGS analysis

Arterial tissue specimens were snap-frozen and stored in liquid nitrogen. DNA was extracted using the Epicentre MasterPure^TM^ DNA Purification Kit according to the manufacturer’s instructions (Epicentre, Madison, WI, USA). The HOMI*NGS* technology was used to enable the identification of nearly 600 oral bacterial species, as previously described [21–23]. Briefly, 16S rRNA genes were amplified (V3–V4 hypervariable region) and processed using a modified MiSeq (Illumina, Inc., San Diego, CA, USA) platform, as described by Caporaso et al. [24]. Species taxa identification and frequency were determined using the ProbeSeq program [22,23]. Sequence reads that were uniquely electronically hybridized to one species- or one genus-specific probe were counted as a ‘hit’ and accumulated. Genus probes can provide an estimate of (1) unidentified oral and non-oral species, or (2) the presence of genera of known species for which a specific species probe within ProbeSeq against the V3–V4 16S rRNA gene sequence has not been designed. More than 20% of unmatched reads is reflective of significant bacterial DNA degradation if proportional loss of bacterial identification can be determined (i.e. increased underrepresentation of species or genera hits as the amount of unaccounted reads increases). Total hits by species or genus probe by patient were used for statistical analyses.

### Statistical analyses

Shannon and Simpson alpha diversity indexes were determined using an online open source program (http://www.comparingpartitions.info/index.php?link=Tool). Differences between groups (i.e. coronary vs. femoral, male vs. female, male/female patients with younger than 60 years of age vs. older than 60 years of age) were analyzed using the Mann–Whitney *U*-test with adjustment for differences in sample sizes, using an online tool (http://www.Vassarstats. net/utest.html). PERMANOVA (unrestricted permutation of raw data, 999 permutations, type III partial sum of squares) in the PRIMER 7 program (PRIMER-E Ltd., Ivybridge, UK) was used to compare beta diversity between groups based on Bray–Curtis similarity matrixes derived from bacterial species and genera abundance data (*p *< 0.05). Spearman correlations of bacterial species or genus hits with unmatched reads were determined using an online open source program (http://www.socscistatistics.com/tests/spearman/Default2.aspx; *p *< 0.05).

## Results

Demographic data and clinical characteristics for the 42-patient cohort (*n* = 31 males, *n* = 11 females) are presented in Table 1.

HOMI*NGS* initial aggregate raw data analysis showed that the percentage of sequence reads that e-hybridized to species probes ranged from 5.7% to 84.1% and e-hybridized to genus probes from 1.0% to 65.7% of the total reads by patient (Table 2). The frequency of unmatched reads ranged from 5.9% to 83.1% (*M* [*SD*] = 29,091.7 [20,309.9]; Table 2), reflective of the presence of (1) non-oral bacterial species, (2) novel oral species for which ProbeSeq probes have not been developed, or (3) low-quality reads, possibly resulting from bacterial DNA degradation. HOMI*NGS* data obtained for coronary (*n* = 32) and femoral (*n* = 10) artery tissues showed the presence of 230 and 118 oral bacterial species in these tissues, respectively, with 103 species in common (Table 3). In addition, unknown species detected by genus-specific probes represented 45 and 30 genera for the coronary and femoral artery tissues, respectively (Table 3). Overall, 95 genera (i.e. the sum of 86 genera detected by species probes and an additional nine detected by genus probes) and 245 species were uniquely present in coronary and femoral arteries combined (Table 3).

**Table 2.** HOMI*NGS* aggregate raw data obtained from clinically non-atherosclerotic coronary and femoral artery tissue samples (*n* = 42).

Reads category	Total	Range	Median	Mean	Standard deviation	Mean relative abundance^a^(%)	Range relative abundance^b^ (%)
Species Probes hits	1,490,502	4,782–111,251	28,234	35,488.1	27,572.4	39.79	5.7–84.1
Genus Probes hits	1,033,630	967–86,927	21,326	24,610.2	17,385.1	27.59	1.0–65.7
Unmatched reads	1,221,851	4,754–78,298	21,700	29,091.7	20,309.9	32.62	5.9–83.1

The HOMI*NGS* species identification process is based on an iterative *in silico* hybridization process. Within ProbeSeq program, each 16S rRNA bacterial gene sequence read is matched *in silico* against each unique species probe. A perfect species probe match is recorded as one ‘hit’. Following the species probe matching process, as yet unmatched reads are matched to genus probes. Sequence reads matching neither a species nor genus probe are, finally, accumulated as ‘Unmatched Reads’.

Calculations were based on all patient samples (n=42), *i.e.*, coronary (n=32) or femoral (n=10); ^a^ Mean Relative Abundance is shown as percentage based on the ratio mean/total hits or reads; ^b^ Relative Abundance Range is shown as percentage based on the ratio minimum or maximum hits or reads/total hits or reads, respectively.

The top three unique species among 245 detected in coronary and femoral arteries combined were (in decreasing order of abundance) *P. gingivalis*, *Enterococcus faecalis*, and *Finegoldia magna* (Table 4), while the top three genera among the 95 detected were *Porphyromonas*, *Escherichia*, and *Staphylococcus* (Table 5). In addition, the top five most abundant genera (i.e. *Porphyromonas, Escherichia*, *Staphylococcus, Pseudomonas*, and *Streptococcus*) were consistently detected across all patients (*n* = 42), altogether representing 88.5% of the total hits (Table 5). The taxa *P. gingivalis*, *Rothia mucilaginosa*, *Escherichia*, *Staphylococcus*, *Pseudomonas*, *Streptococcus*, and *Granulicatella* were detected in all patient samples (*n* = 42; Tables 4 and 5). The rest of the taxa exhibited relatively scarce data with occasional high outlier values. The number of hits across patients for these five most abundant genera correlated negatively with their respective number of unmatched reads (data not shown). All *r* correlation values determined by Spearman’s rank correlation were negative (mostly *p *< 0.05), indicating that a significant amount of unmatched reads most likely corresponds to low-quality sequences, leading to stochastic proportional underrepresentation of taxa hits. Although there was no abundance data correlation found between the five most abundant genera in pairwise comparisons, no correlation analyses were performed with any of the clinical variables. Indeed, such correlations were deemed unwarranted or coincidental due to the significant impact by unaccounted low quality reads on absolute total hits.

**Table 3.** Species and genera detection data.

	Coronary tissue	Femoral tissue	Common coronary and femoral	Unique coronary and femoral
Species and/or genus probe-based detection	(*n* = 32)	(*n* = 10)	(*n* = 42)	(*n* = 42)
Species count per species probes	230	118	(103)**^#^**	245
Genera count per genus probes	45	30	(28)**^#^**	47
Genera count per species probes	84	58	(56)**^#^**	86
Genera count unique to genus probes	8	7	(6)**^#^**	9
Total genera count	92	65	(62)**^#^**	95

A species or genus is considered detected if at least one hit is accounted for that species or genus. Species probe hits may be combined with corresponding genus probe hits to determine total number of genera detected. ^#^ Number of common species or genera is represented by (number).

**Table 4.** Ten most prominent species and genera identified by HOMI*NGS*.

Species Name	No. positives	Total hits	Hits range	Median hits	Mean hits	Standard deviation	% Relative abundance^a^
1. *Porphyromonas gingivalis*	42	1,180,493	103–101,244	18,523.5	28,107.0	27,625.7	79.20%
2. *Enterococcus faecalis*	32	46,100	0–18,537	1	1,097.6	3,529.0	3.09%
*3. Finegoldia magna*	22	32,795	0–13,180	1	780.8	2,831.9	2.20%
4. *Pseudomonas aeruginosa*	25	30,857	0–7,848	17	734.7	1,445.7	2.07%
5. *Haemophilus parainfluenzae*	19	7,685	0–4,773	0	183.0	765.3	0.52%
6. *Rothia mucilaginosa*	42	6,675	17–2,088	45	158.9	374.2	0.45%
7. *Gemella haemolysans*	33	6,609	0–5,932	1.5	157.4	916.2	0.44%
8. *Brevundimonas diminuta*	8	6,560	0–3,324	0	156.2	562.1	0.44%
9. *Stenotrophomonasmaltophilia*	11	4,800	0–2,845	0	114.3	500.7	0.32%
10. *Gemella morbillorum*	16	4,369	0–4,094	0	104.0	631.4	0.29%

Calculations were based on all patient samples (n=42), *i.e.*, coronary (n=32) or femoral (n=10); ^a^ Mean Relative Abundance is shown as percentage based on the ratio mean/total hits per total 245 species detected (1,490,502 hits).

**Table 5.** Most prominent genera.

Genus name	No. positives	Total hits	Hits range	Median hits	Mean hits	Standard deviation	% Relative abundance^a^
1. *Porphyromonas*	42	1,180,564	103–101,245	18,523.5	28,108.7	27,625.3	46.77%
2. *Escherichia*	42	488,103	112–83,578	4,351.5	11,621.5	18,261.4	19.34%
3. *Staphylococcus*	42	264,248	11–43,098	3,688.5	6,291.6	8,475.1	10.47%
4. *Pseudomonas*	42	194,684	8–26,997	2,280.5	4,635.3	6,010.3	7.71%
5. *Streptococcus*	42	105,195	52–19,802	577	2,504.6	4,226.8	4.17%
6. *Enterococcus*	32	46,765	0–18,537	1	1,113.5	3,525.4	1.85%
7. *Acinetobacter*	39	46,382	0–32,132	4	1,104.3	4,982.6	1.84%
8. *Finegoldia*	22	32,795	0–13,180	1	780.8	2,831.9	1.30%
9. *Anaerococcus*	20	25,337	0–12,884	0	603.3	2,096.5	1.00%
10. *Granulicatella*	42	20,038	7–13,066	18	477.1	2,157.4	0.79%

Calculations were based on all patient samples (n=42), *i.e.*, coronary (n=32) or femoral (n=10); ^a^ Mean Relative Abundance is shown as percentage based on the ratio mean/total hits per total 95 genera detected (2,524,132 hits).

**Figure 1.**
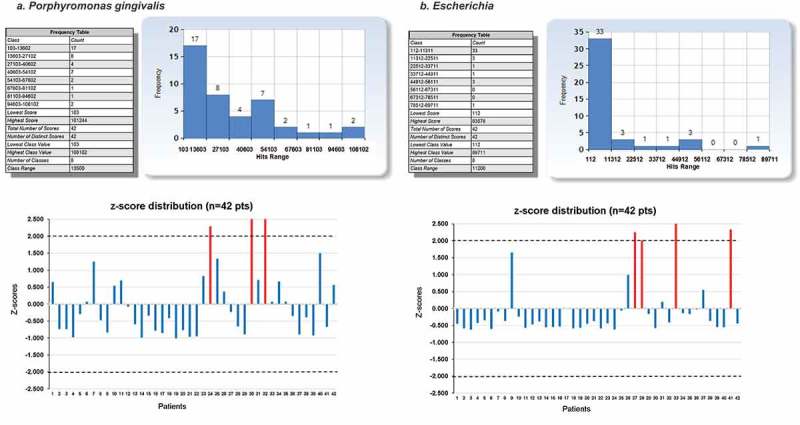
Abundance frequency plots and *z*-score distribution for species *P*orphyromonas *gingivalis* and genus *Escherichia* in clinically non-atherosclerotic coronary and femoral artery tissues (a) *P*. *gingivalis* (b) Genus *Escherichia*. Frequency distributions for most abundant taxa, *P. gingivalis* (a) and genus *Escherichia* (b), are shown. Both taxa were detected in significant amounts in virtually all coronary and femoral artery tissue patient samples (*n* = 42). The distributions of both taxa were similar. However, the *z*-score distributions showed no outliers overlap (red bars), i.e. patients 24, 30, and 32 for *P. gingivalis* (*z*-score plot in (a)) and patients 27, 28, 33, and 41 for *Escherichia* (*z*-score plot in (b)). Thus, *P. gingivalis* abundance data do not correlate with those of *Escherichia* (*r* = 0.02552; *p* = 0.87252).

Also, no significant differences were found in bacterial alpha or beta diversity measurement between coronary and femoral artery tissues (*p *≥ 0.05). Therefore, the data were pooled for further analysis. In addition, no significant differences were found in alpha or beta diversity between males (*n* = 31) and females (*n* = 11), or between patients age 60 or younger (*n* = 15) and those older than 60 years of age (*n* = 27; *p *≥ 0.05; data not shown). However, despite the fact that there were no differences in alpha and beta diversity between the above-mentioned subgroups, *P. gingivalis* and the genus *Escherichia* were the most abundant taxa. These two taxa had similar hits per patient distributions in combined coronary and femoral artery tissues data sets (Figure 1(a) and (b)). However, *P. gingivalis* was significantly more abundant (*p *= 0.0005; Figure 1(a) and (b)). *P. gingivalis* alone accounted for 79.2% of the total species probe hits (Table 4), and genus *Porphyromonas* accounted for 46.8% of the total combined species and genus probe hits (Table 5). The second most abundant genus, *Escherichia*, for which no *E. coli* probe had been designed, accounted for 19.3% of total combined species and genus probe hits. The *z*-score distributions showed that patient outliers with regard to the abundance data for these two taxa were different (Figure 1(a) and (b)). The potential implications of these results are discussed below.

## Discussion

This is the first study to report using HOMI*NGS* targeted metagenomics to screen for nearly 600 known oral bacterial species in artery tissues clinically unaffected with atherosclerosis that were obtained from patients with atherosclerotic CVD who underwent coronary or femoral artery bypass surgery. *P. gingivalis* was by far the most abundant species, representing nearly 80% of all bacterial species counts.

Three other species (i.e. *E. faecalis*, *F. magna*, and *P. aeruginosa*) were detected with a relative abundance of 2% to just over 3% compared with all remaining species detected at <1%. All three species have been identified as pathogens involved in cardiovascular complications. For instance, *E. faecalis* is associated with infective endocarditis [25,26]. *F. magna* has been shown to invade skin cells [21], and is one of the most frequent pathogens in the etiology of postoperative and prosthetic implant-related infection [22]. It is also associated with mediastinitis following coronary artery bypass [23]. *F. magna* has been detected in rare cases in atherosclerotic plaques from carotid arteries [27]. Finally, chronic *P. aeruginosa* infection has been shown to increase aorta and coronary artery thickness and wall rigidity in a rat model of atherosclerosis [28].

In this study, the genus *Escherichia* was the second most abundant behind *Porphyromonas*, while relative abundances of both taxa did not correlate across patients. No species probes were available within the HOMI*NGS* platform to ascertain species identification within the *Escherichia* genus. Nevertheless, such findings would indicate detection of taxa of non-oral origin. *Staphylococcus* was the third most abundant genus detected, with the dual species probe for *S. aureus* and *S. gallinarum* accounting for nearly 27% of the total hits for this genus in 41/42 patients, respectively. *Staphylococcus* spp. have been detected in atherosclerotic plaques of patients with coronary heart disease [16]. Although HOMI*NGS* focuses mainly on oral taxa, approximately 70–90% of the taxa were accounted for, with significant detection at the genus level by species and genus probes. Unmatched reads could correspond to species that are exclusively of non-oral origin, strain variation, or sequencing error.

The present results differ from previous metagenomics studies that focused on the analysis of atherosclerotic plaque and did not find *P. gingivalis* as a predominant species [15–19]. These studies did not use clinically non-atherosclerotic arterial walls from patients diagnosed with atherosclerosis as control tissues. For instance, Ott et al. [16] used control coronary artery tissues from patients who died from malignancies and used tissues from heart-beating tissue donors, both without presence of atherosclerosis of any coronary artery per macroscopic examination [16]. The authors did not, however, detect bacterial DNA in the control tissues as opposed to atherosclerotic plaque samples, and are hence in contrast with the current findings.

In addition, since the present study used clinically healthy tissues from patients with atherosclerosis, the results differ significantly from those obtained from atherosclerotic plaque material through metagenomic approaches [15–19]. These studies showed little overlap at the species level regarding the identified predominant taxa. Also, as results are inherently dependent on the technological platform used, reproducibility needs to be assessed using complementary approaches in larger cohorts. While the use of HOMI*NGS* has enabled a unique bacterial profile to be identified of clinically non-atherosclerotic artery tissues for oral species, future investigations will benefit from alternative approaches such as the Pathochip array technology or sequencing algorithms that allow for comprehensive analysis of all bacterial taxa [29,30].

Although the periodontal disease status of our patient population is unknown, C-reactive protein (CRP) levels recorded for 17/42 patients ranged from 27 to 91 mg/L (Table 1), which could possibly correlate with periodontal disease in patients with cardiovascular disease [8]. Overall, the significant detection of *P. gingivalis* in the arterial tissues of all patients (*n* = 42) in this study is in agreement with other studies, implicating *P. gingivalis* in the development of atherosclerosis. However, *A. actinomycetemcomitans* was not detected, except in one sample with 11 hits, which might reflect fundamental colonization differences between plaque material and clinically non-atherosclerotic arterial tissues. Similarly, *Chlamydia pneumonia* was detected in four patients at very low levels.

A large body of evidence has implicated the periodontal pathogen *P. gingivalis* in atherosclerosis [11]. However, the role of *P. gingivalis* as an etiological agent of atherosclerosis is yet to be elucidated. *P. gingivali*s was shown to reside in diseased atherosclerotic tissues and the aneurysmal wall of blood vessels and to induce atherosclerosis in pigs following bacteremia regardless of cholesterol level [16,31]. It has been suggested that periodontal disease predisposes for atherosclerosis due to an association between the presence of antibodies against *P. gingivalis* and atherosclerosis in large cohort studies [3,4,7,8,32]. Based on epidemiological and experimental studies, several mechanisms have been proposed [7,8,10]: (1) intracellular invasion of arteries at the sites of atherosclerotic disease by periodontal pathogens [10]; (2) increased responsiveness of the immune response to the presence of *P. gingivalis*, with possible involvement of proinflammatory bacterial products such as lipopolysaccharides or phosphorylated dihydroceramides [7,33,34]; and (3) cross-reactivity of antibacterial heat-shock protein antibodies with human heat-shock proteins [7,8].

Overall, the vastly predominant presence of *P. gingivalis* in clinically healthy arteries observed in this study is in line with the concept that *P. gingivalis* possesses unique properties to invade the arterial walls to survive intracellularly and escape the immune system [28]. It is unknown, however, whether the bacterial colonization of healthy tissues occurs prior to atherosclerosis disease onset or whether diseased tissues form before the colonization, thereby enabling subsequent colonization. It is also unknown for how long bacteria remain alive within tissue, and which species act as bystanders or contribute to the development of atherosclerosis in the initial stages of the disease. Longitudinal studies in animal models for atherosclerosis will be required to identify biomarkers useful in human studies for the determination of whether inflammation precedes or follows bacterial colonization of damaged or non-damaged tissues and how colonization evolves during the course of the disease. Understanding of the different stages of colonization could provide some explanation as to why certain species would be present in clinically ‘healthy’ tissues and absent in atherosclerotic plaque. In particular, investigation of mechanisms of periodontal disease that would influence such distant site colonization will be necessary to develop improved prevention and therapies for atherosclerosis [35,36].

In conclusion, this study provides insight into the presence and types of bacteria found in clinically healthy artery tissue. These species may be associated with the initiation and/or exacerbation of atherosclerosis, with or without any role in causation. The role of *P. gingivalis*, the most predominant species identified in this study, and other oral bacteria potentially involved in the disease process may be further elucidated in future studies.
